# Mathematical solutions in internal dose assessment: A comparison of Python-based differential equation solvers in biokinetic modeling

**DOI:** 10.1088/1361-6498/ad0409

**Published:** 2023-10-30

**Authors:** Emmanuel Matey Mate-Kole, Dmitri Margot, Shaheen Azim Dewji

**Affiliations:** 1 Nuclear and Radiological Engineering and Medical Physics Programs, Georgia Institute of Technology, Atlanta, GA, United States of America

**Keywords:** internal dosimetry, ODE solvers, Python, compartmental analysis, biokinetic modeling

## Abstract

In biokinetic modeling systems employed for radiation protection, biological retention and excretion have been modeled as a series of discretized compartments representing the organs and tissues of the human body. Fractional retention and excretion in these organ and tissue systems have been mathematically governed by a series of coupled first-order ordinary differential equations (ODEs). The coupled ODE systems comprising the biokinetic models are usually stiff due to the severe difference between rapid and slow transfers between compartments. In this study, the capabilities of solving a complex coupled system of ODEs for biokinetic modeling were evaluated by comparing different Python programming language solvers and solving methods with the motivation of establishing a framework that enables multi-level analysis. The stability of the solvers was analyzed to select the best performers for solving the biokinetic problems. A Python-based linear algebraic method was also explored to examine how the numerical methods deviated from an analytical or semi-analytical method. Results demonstrated that customized implicit methods resulted in an enhanced stable solution for the inhaled ^60^Co (Type M) and ^131^I (Type F) exposure scenarios for the inhalation pathway of the International Commission on Radiological Protection (ICRP) Publication 130 Human Respiratory Tract Model (HRTM). The customized implementation of the Python-based implicit solvers resulted in approximately consistent solutions with the Python-based matrix exponential method (*expm*). The differences generally observed between the implicit solvers and *expm* are attributable to numerical precision and the order of numerical approximation of the numerical solvers. This study provides the first analysis of a list of Python ODE solvers and methods by comparing their usage for solving biokinetic models using the ICRP Publication 130 HRTM and provides a framework for the selection of the most appropriate ODE solvers and methods in Python language to implement for modeling the distribution of internal radioactivity.

## 1. Introduction

A biokinetic model describes the uptake, retention, and clearance of incorporated radionuclides in a biological system using mathematical formalisms defined as a function of intake pathways. Due to the complexity of biological systems, the biokinetic models are classified based on the specific intake scenario (Nosske *et al* 2011, Li 2018, Zhang *et al* 2021):

• **Human Respiratory Tract Model (HRTM):** Describes the deposition of inhaled radionuclides in the respiratory tract, mechanical clearance to other regions of the respiratory tract and the alimentary tract, and absorption into blood;
• **Human Alimentary Tract Model (HATM):** Quantifies the intake of radionuclides by ingestion, absorption into blood, and clearance (excretion) through feces;
• **Wound Model:** Describes radionuclide uptake, retention, and clearance through wound contamination with subsequent absorption to blood; and
• **Systemic Model:** Describes the systemic circulation of radionuclides after entering the blood through injection (either accidental injection or radiopharmaceutical applications) and the aforementioned internal exposure pathways.

Prior to the mid-1960s, knowledge of the quantification of internally incorporated radionuclides was limited (Stather 2004). However, the mandate to increase knowledge of radiation monitoring and prevention of undue exposure was improved as a result of the establishment of organizations, among which was the International Commission on Radiological Protection (ICRP). Building on the foundation laid by the first ICRP biokinetic models for assessing radionuclide intake and dose in the body (ICRP 1959), significant advancements have been realized for prospective and retrospective radiological protection over the years. To this effort, the framework of a compartment-based method found much applicability for use in internal dosimetry applications (Sanchez 2005). The compartmental analysis applied to biological systems separates the organs and tissues of the body into several individual compartments, which are connected through the metabolic exchange of materials (i.e. internalized radionuclides) (Vicini and Brill 2008). The system of compartments is mathematically modeled as a system of coupled ordinary differential equations (ODEs). This mathematical concept describes a state of evolution, given that the state variable changes over time. The conformity of the system of compartments to an ODE allows for its applicability in chemical reactions studies, drug kinetics in pharmacology, and metabolic systems studies (Anderson 1983, Aro 1996, Postawa *et al* 2020).

Moreover, due to the complexity of systems constituting biokinetic models, computational capabilities were harnessed for solving the coupled system and have been pervasively applied in internal dosimetry (Killough and Eckerman 1984, Birchall and James 1989, Sadre Momtaz and Sheikhangafsheh 2021). Several solving methods, algorithms, and computer codes have been developed for solving general kinetic problems, including biokinetics (Dunford and Johnson 1987, French *et al* 1988, Strenge *et al* 1990, Eckerman *et al* 2006, Fell *et al* 2007, Bertelli *et al* 2008). However, most of these codes and tools are based on routine programming languages lacking modern dynamic capabilities, despite the flexibility of symbolic representations in coding syntax and dynamic memory. As new operating system versions have evolved and new models have been developed, migrating toolkits across platforms cannot be assumed to be a simple translation and compilation process (Stabin *et al* 2005). Moreover, open-source toolkits for internal dose assessment are limited, if available, and functionalities are even further limited. Expansions of enhanced and customizable solution tools have necessitated developing compartmental models in Python codified form with an enhanced coupling capability.

The objective of this study was to test the capabilities of different solvers and methods in the modern programming language Python for solving an extensive coupled system of ODEs for biokinetic modeling. Python is an interpreted and dynamic programming language that offers flexibility by supporting an agile development process (Schmitt *et al* 2022). The choice to explore Python capabilities for solving the biokinetic models was due to the existence of its direct interfacing potential with a multilevel parallel object-oriented framework for an optimized parametric analysis–a promising platform for the interoperation of disparate codes, modules, and tools.

Metabolic models are divided into several compartments, which are solved as systems of ODEs to estimate the biodistribution and retention of the radioactive material in the body (ICRP 1972). However, the coupled system for a given radionuclide and clearance class could comprise several rapidly and slowly varying components resulting in a problem known as stiffness. Stiffness has been identified as a prominent challenge inherent to the complexities of biokinetic modeling. Stiff systems can result in significant numerical instabilities if not solved with the appropriate solvers and methods, where stability implies solutions that do not exhibit oscillating derivatives, inaccurate solutions (including negative solutions) or divergent solutions (a solution that happens to be unusually large positive or large negative), and solutions with amplification factor less than or equal to one over time (indicating growth in error with time as the solution evolves) (Yano *et al* 2012). This study conducted a careful analysis of standard ODE solvers and solving methods in Python, defining the application regime for Python solvers based on their applicability to biokinetic modeling. As such, this study’s framing elaborates on the problem challenge and stiffness quantification.

This study focuses on the methodological approach adopted and recommended by ICRP (1994, 1995, 1996, 2006, 2007, 2015, 2016a, 2016b, 2017) for internal dose assessment by reconstructing the biokinetic models into Python code and solving the system with different Python numerical solvers through practical examples. The radionuclides explored in this study were selected to demonstrate the solving capabilities of Python-based ODE solvers and solving methods, which are ^60^Co (moderate clearing–Type M) and ^131^I (fast clearing–Type F). Both ^60^Co (Type M) and ^131^I (Type F) were chosen to encapsulate rapidly and slowly varying components to indicate the level of stiffness of the differential equations posed by the biokinetic models. The choice for a moderate and rapid clearing radionuclide is necessary to demonstrate the tolerance level of the solvers for both the HTRM and systemic biokinetic model. These solvers and solving methods can be comprehensively applied and adapted for solving any other element-specific biokinetic modeling problem. Cobalt, in general, can be encountered in various chemical forms, including metal dust, oxides, nitrates, and chlorides (ICRP 2017). Cobalt-60 is of concern for emergency public protection primarily due to its potential for use in radiological dispersal devices, where inhalation is considered the most probable mode of intake from internal exposures (Dewji *et al* 2013). Conversely, iodine may be encountered in industry in various chemical and physical forms, including vapors and gases, organic compounds, and particulate forms (ICRP 2017). Iodine-131 is associated with nuclear power plant effluent from the release of fission products (DJurović *et al* 2016, Sulaiman *et al* 2018) or an improvised nuclear device (Marcus *et al* 2006, Chen and Tenforde 2010, Pan and Ungar 2012). Practical examples given in this paper for demonstration employed the ICRP HRTM given in Publication 130 (ICRP 2015); however, the fundamental mathematical approach governing the estimation of activity burden in the body remains consistent and could be adopted for other internal exposure scenarios of concern.

## 2. Methodology

The methodology applied in this study first addressed the framework of compartment-based biokinetic models, from which different mathematical solutions of ODEs in Python were compared and analyzed for stability. Both explicit and implicit methods, an optimized implicit method, and an algebraic methodology, such as matrix exponentiation, were compared. The biokinetic solutions obtained using the exploited Python solvers and the solving methods were compared further with the biokinetic retention solutions from the ICRP Occupational Intake of Radionuclides (OIR) Electronic Annex Distribution Set to quantify the agreement with published data ICRP (2015, 2016b, 2017). The solvers with the most stable solutions were then recommended for further development of a robust computational framework.

### 2.1. ODE form of biokinetic systems

The dynamic behavior of an inhaled radionuclide in the human respiratory tract is described in ICRP Publication 130 (ICRP 2015). The ICRP Publication 130 model is the updated form of the previous HRTM defined in Publication 66 (ICRP 1994). From the time of intake of a radionuclide, the rate of exchange of the material concentration framed as a mathematical description of the evolution of radionuclides in the body is represented in its general form in equation (1) (ICRP 2015, Issa and Serge 2021, Zhang *et al* 2021):

\begin{equation*}\frac{{{\text{d}}{A_{i,j}}\left( t \right)}}{{{\text{d}}t}} = \mathop \sum \limits_{\begin{array}{*{20}{c}} {k = 1} \\ {k \ne j} \end{array}}^M {A_{i,k}}{\lambda _{i,k,j}} - {A_{i,j}}\left[ {\mathop \sum \limits_{\begin{array}{*{20}{c}} {k = 1} \\ {k \ne j} \end{array}}^M {\lambda _{i,k,j}} + \lambda _i^{\text{D}}} \right] + \mathop \sum \limits_{k = 1}^{i - 1} {A_{k,j}}{\beta _{k,i}}\lambda _{i\,\,}^{\text{D}}\end{equation*}

where *M* is the number of compartments describing the kinetics;

${\lambda _{i,k,j}}$ is the fractional transfer rate of chain member *i,* from donor compartment *j* to receiver compartment *k* in the biokinetic model;

$\lambda _i^{\text{D}}$ is the physical decay constant of decay chain member *i;* and

${\beta _{k,i}}$ is the fraction of decays of decay chain member *k* forming *i.*

${A_{i,j}}\left( t \right)$ is the activity (*Bq*) of the radionuclide *i* in compartment *j* at time *t.*

Thus, equation (1) serves as the governing equation for radionuclide and its progeny’s distribution in the body conforming to a system first-order ordinary differential equation. With the initial conditions for each compartment (${A_{i,j}}\left( 0 \right)$), the system of ODEs can be solved using applicable numerical/algebraic methods. ICRP Publication 130 (2015) further details the framework for utilizing biokinetic models to develop internal dose coefficients.

### 2.2. ODE solvers and solving methods

First, solvers in the *solve_ivp* package from Python Scipy scientific library (Christensen *et al* 2015, Virtanen *et al* 2020) were used to solve the ODE system. The *solve_ivp* package is a condensed solver comprising six solvers (table 1) divided into stiff and non-stiff solvers, where LSODA is a solver known for its universal adaptation.

**Table 1.** Python language *solve_ivp* ODE solvers with their respective methods (Hagen and Mayorov 2019).

Method	Solver	Recommended choice of ODE problems
Explicit	RK45	Non-stiff
	RK23	Non-stiff
	DOP853	Non-stiff
Implicit	Radau	Stiff
	BDF	Stiff
Universal choice	LSODA	Universal

In general, ODEs can be classified as non-stiff and stiff based on the problem being modeled. Non-stiff problems are problems whereby all the components evolve simultaneously, while stiff problems are classified as problems with rapidly and slowly varying components; these generally arise in biokinetic modeling of the inhalation pathway (Byrne and Hindmarsh 1987, Omale *et al* 2014). A stiff problem, typically encountered in internal dosimetry, is known to benefit from analytical or semi-analytical approaches such as eigenvalue methods, where analytical expressions are derived numerically (Killough and Eckerman 1984, Birchall and James 1989, Fell *et al* 2007). The benefit of a numerical method is derived from its versatility and is, therefore, better adapted to tackle non-linearities (Fell *et al* 2007). The numerical solvers and methods employed in this work are discussed as follows. The *solve_ivp* function is part of a class-based package of explicit and implicit ODE solvers in SciPy for solving standard initial value problems (Hagen and Mayorov 2019). The function in SciPy can be called by default using *scipy.integrate.solve_ivp(fun, t_span, y*0, method = ‘RK45’, t_eval = None, dense_output = False, events = None, vectorized = False, args = None, **options), where

• *fun* defines the right-hand side of the equations, which has a calling signature as *func(t,y)*;
• *t_span* defines the time interval of integration (0*,tf);*
• *y*0 defines the initial conditions or an array like the initial state of the system, method specify the solving method to implement;
• *t_eval* defines the time for which the solutions are stored;
• *dense_output* specifies whether to implement a continuous solution;
• *events* tracks events that applies termination if that specific event occurs or redirect computation based on the direction of zero crossing;
• *vectorized* parameter specifies if *fun* is vectorized;
• *args* are arguments to pass to user-defined functions; and
• **options include
  ∘ *first_step*—initial step size;
  ∘ *max_step*—maximum step size;
  ∘ *rtol* and *atol*—relative and absolute tolerances that ensure local error control by keeping the local error estimate less than *atol + rtol × abs(y)*;
  ∘ *jac*—Jacobian matrix/array;
  ∘ *jac_sparsity*—defines the sparsity structure of the Jacobian matrix for a finite-difference approximation;
  ∘ *lband*—defines the bandwidth of the Jacobian for the LSODA method;
  ∘ *uband*—defines bandwidth of the Jacobian for the LSODA method; and
  ∘ *min_step*—defines the minimum allowed step size for the LSODA method.

Methods explored in this work were RK45, RK23, DOP853, Radau, BDF, and LSODA.

For each of the biokinetic models, matrices of constant coefficients (sparse matrix in most cases) were defined, and the right-hand side of the ODEs was further expressed as matrix multiplications. The matrix multiplications were then fed into fun in the *solve_ivp* function. Implementing the default parameters with each of the methods solutions over a 50 year period were obtained for a single acute unit intake of activity in the respiratory tract. The default implementations include the following:

1. Step sizes were allowed to be chosen by the algorithm.
2. By default, relative and absolute tolerances (*rtol* and *atol*) utilized are 10^−3^ and 10^−6^, respectively.
3. Jacobian was approximated using the finite difference method.

The optional function parameters *of solve_ivp*, such as *rtol, atol*, and time step (*max_step*), were further adjusted to exercise strict error control. The *rtol* and *atol* values were reduced to 10^−12^ and 10^−15^, respectively, and the function was reevaluated. Based on the stiffness level of the ^131^I scenario, the maximum step size was restricted to 0.2, in addition to *rtol* and *atol* of 10^−12^ and 10^−15^. The label for restricting the step size, in addition to the reduced tolerances, was additionally customized. The biokinetic model problem sets were solved for each case discussed, and the activity retained in each compartment (for the specific radionuclide) was tracked.

#### 2.2.1. Explicit solvers

According to SciPy, the RK45 method, which is the default method for *solve_ivp*, is an explicit Runge–Kutta method of order 5(4) (Dormand and Prince 1980). The RK45 method controls the error by assuming the accuracy of the fourth-order method, but steps are taken using fifth-order local extrapolation. The method utilizes a quartic interpolation polynomial–a fourth-degree polynomial (Shampine 1986, Hairer and Ostermann 1990) and can be applied to a complex domain–applicable to the other explicit methods investigated. RK23 is another explicit Runge–Kutta method of order 3(2), where in contrast to RK45, the error is controlled by assuming the accuracy of the second-order method and third-order local interpolation method is utilized by the steps taken (Bogacki and Shampine 1989). When computing continuous solutions, RK23 uses a cubic Hermite polynomial for its dense output. The last solely explicit solver/method exploited was DOP853 of order 8, which is a Python implementation of the DOP853 algorithm initially written in FORTRAN (Hairer *et al* 1993).

#### 2.2.2. Implicit solvers

The next category of solving methods investigated was implicit methods. Radau is an implicit method of the Radau IIA family of order 5 (Wanner and Hairer 1996). To satisfy the collocation condition in the problem domain, Radau employs cubic polynomials for dense output. Additionally, Radau IIA methods are considered a one-step collocation-type method, which is approximated by polynomials for which the stability of a stiff differential equation is tested by utilizing sub-diagonal Padé approximations (Hairer and Wanner 2015). For the BDF method, a multi-step variable order (1–5) based on backward differentiation formula is employed, for which accuracy is enhanced using the numerical differentiation formulas modification (Shampine and Reichelt 1997) and can be applied to complex domains.

#### 2.2.3. Universal solvers

The final method investigated was LSODA. LSODA is a FORTRAN wrapper from ODEPACK, which is equipped with automatic stiffness detection capability. ODEPACK is a collection of FORTRAN solvers for standard initial value ODE problems (Hindmarsh 1983). LSODA is usually considered a universal choice, and its algorithm permits switching between Adams (a method for non-stiff systems) and BDF (a method suitable for stiff systems of equations) (Petzold 1983). Moreover, some problem types may still need to be fully classified or understood. In this case, SciPy recommends using RK45; if it results in unusually many iterations (diverge or fail), the problem under study is probably stiff and thus should be approached with Radau or BDF (Virtanen *et al* 2020).

#### 2.2.4. Matrix exponential methods

Matrix theory is considered to be very important for finding the solution to a system of linear ODEs (Bellman 1997). A study published by Birchall and James (1989) described a microcomputer algorithm for solving first-order compartmental models using matrix algebra by essentially finding the exponential of the matrix of constant coefficients. The study by Birchall and James (1989) highlighted some difficulties with existing methodologies for matrix exponentiation evaluation, such as the complexity of evaluation through eigenvalue decomposition and for non-symmetric matrices. Earlier publications also made a major assumption for using eigenvalue methods for relatively simple compartmental models (Killough and Eckerman 1984). According to Killough and Eckerman (1984), to solve an initial value problem (IVP) of the form

\begin{equation*}\frac{{{\text{d}}Y}}{{{\text{d}}t}} = \,AY\end{equation*}

\begin{equation*}Y\left( 0 \right) = \,{Y^{\,0}}\end{equation*}

where $Y$ is a vector of *N* functions;

$A$ is a constant *N* × *N* matrix coefficient; and

${Y^{\,0}}$ is a vector of initial values of $Y$.

It was assumed that the eigenvectors form a linearly independent set. An eigenvalue is a particular set of scalars, which are also known as characteristic roots, connected with a linear system of equations. An eigenvector is a non-zero vector in linear algebra that can only change direction when scaled by a characteristic root known as the corresponding eigenvalue (Hoffman and Kunze 1971). However, the eigenvalue approach breaks down for cases when the system contains degenerate compartments, defined as consecutive compartments with similar transfer rates. A solution to curb this failure is to perturb the model parameters by a small fraction for the perturbed system to have linearly independent eigenvectors (Killough and Eckerman 1984). To avoid problems with eigenvalue approaches for analytical evaluation, this work exploited other approaches for matrix exponential. A unique solution to the problem in equation (2) can be denoted as ${{\text{e}}^{At}}$. This matrix exponential which can be defined in a standardized form by power series denoted in equation (4) (Polig 2001, Moler and Van Loan 2003).

\begin{equation*}{{\text{e}}^{At}} = \sum\limits_{i = 0}^\infty \frac{{{{\left( {At} \right)}^i}}}{{i!}}.\end{equation*}

To compute equation (4), Birchall and James (1989) proposed an optimized series expansion approach due to the difficulty encountered in using standard Taylor series expansion. In this study, the matrix exponential method (*expm*), which uses the Padé approximation in Python language, is employed for analytical insight to quantify the deviation of the numerical methods (Al-Mohy and Higham 2010). For an *n × n* matrix A, Padé approximation for a simple case as ${{\text{e}}^A}$ can be expressed as (Arioli *et al* 1996, Sidje 1998, Li *et al* 2011):

\begin{equation*}{R_{pq}}\left( A \right) = \frac{{{N_{pq}}\left( A \right)}}{{{D_{pq}}\left( A \right)}}\,\end{equation*}

where

\begin{equation*}{N_{pq}}\left( A \right) = \mathop \sum \limits_{j = 0}^p \frac{{\left( {p + q - j} \right)!p!}}{{\left( {p + q} \right)!j!\left( {p - j} \right)!}}{A^j}\,\end{equation*}

\begin{equation*}{D_{pq}}\left( A \right) = \mathop \sum \limits_{j = 0}^q \frac{{\left( {p + q - j} \right)!q!}}{{\left( {p + q} \right)!j!\left( {q - j} \right)!}}{\left( { - A} \right)^j}\,\end{equation*}

where $N$ has a degree of $p$; and $D$ has a degree of $q$. The Padé approximation, in this case, can be referred to as (*p,q*) Padé approximation. For ${{\text{e}}^{At}}$, *expm* evaluates the linear system using the analogous form of equation (5) as given in equation (8).

\begin{equation*}{R_{pq}}\left( {At} \right) = \frac{{{N_{pq}}\left( {At} \right)}}{{{D_{pq}}\left( {At} \right)}}\,.\end{equation*}

Additionally, the Python language *expm* function implements a new scaling and squaring algorithm for matrix exponentiation with a variable order that is decided based on the array provided (Al-Mohy and Higham 2010). The benefit of this approach is that it alleviates over-scaling problems by exploiting: (1) the concept of triangularity to ensure that the diagonal and first-off diagonal are computed accurately and (2) a backward error analysis for a refined truncation error bound. The matrix exponential method, derived from matrix theory, is considered advantageous compared to numerical methods because it has been known to provide virtually exact solutions to a system of equations (Ball and Adams 1967). It is worth noting that matrix exponential methods are primarily limited to linear systems of ODEs with some exceptions (Ball and Adams 1967, Moler and Van Loan 2003). Nonetheless, a conscious effort was made to investigate the linear algebraic method in addition to the numerical methods in Python programming language to have a robust and comprehensive computational framework to handle both stability and non-linear problems and have the best efficiency in terms of computational time.

### 2.3. Case studies: ^60^Co and ^131^I ICRP HRTM biokinetic solving methods

According to ICRP Publication 66 (ICRP 1994), the HRTM system is divided into four anatomical regions: the extrathoracic region (ET), comprising of the anterior nose (ET1) and the posterior nasal passages, larynx, pharynx, and mouth (ET2); the bronchial region (BB), consisting of the trachea and bronchi from which deposited material is cleared by ciliary action; the bronchiolar region (bb) consisting of the bronchioles and terminal bronchioles; and lastly the alveolar-interstitial region (AI), consisting of the bronchioles with some alveoli apposed, the alveolar ducts and sacs with their alveoli, and the interstitial connective tissue, with each of these four regions containing lymphatic tissue (LT) or its components. These collectively define the ICRP Publication 66 HRTM. An updated version of the HRTM was reported in the ICRP Publication 130, where the oral passage is no longer included in region ET2 for consistency with the HATM (ICRP 2015). ICRP Publication 130 can be consulted for detailed information regarding the revised HRTM (ICRP 2015).

**Figure 1.**
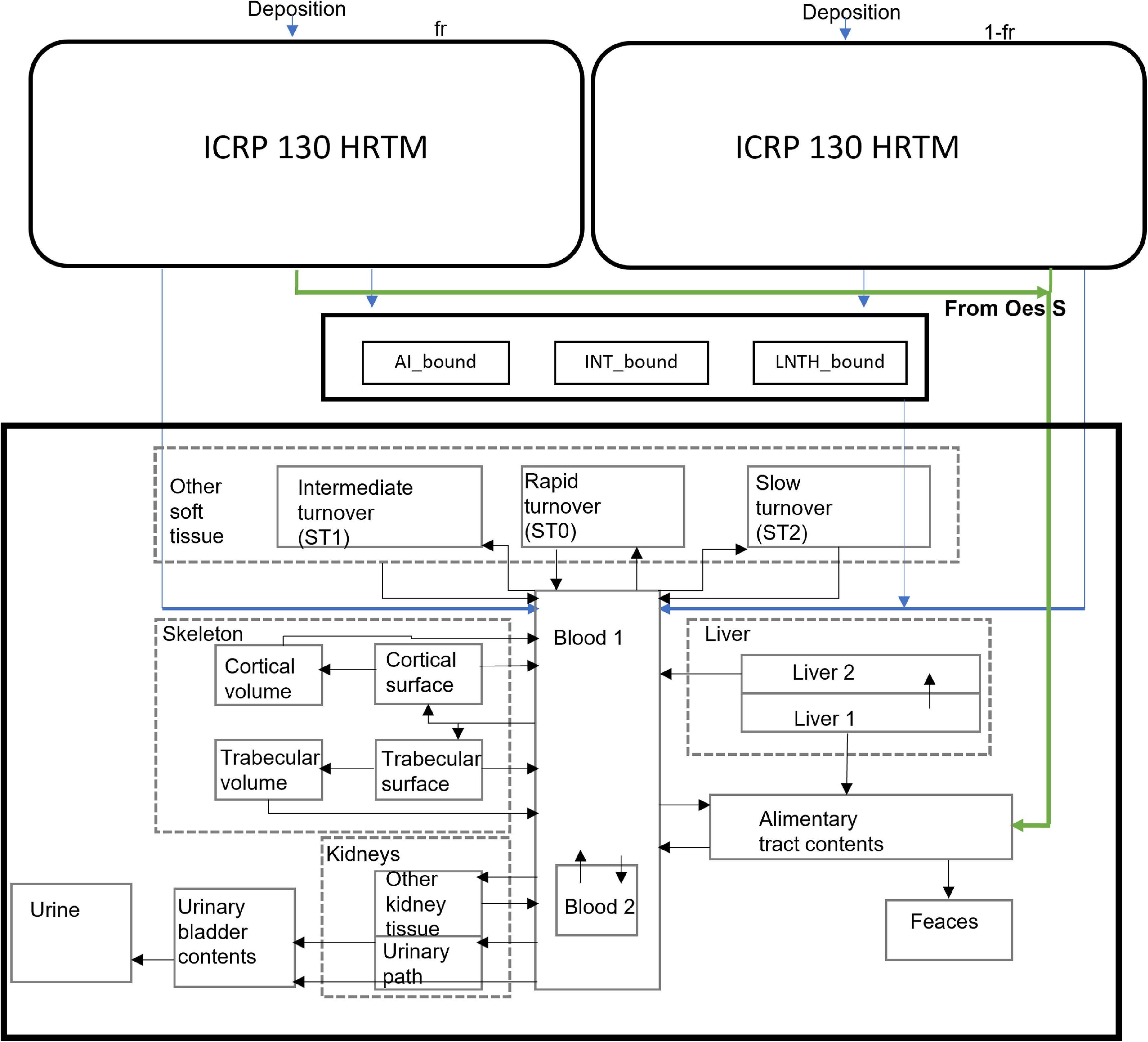
Coupled inhalation compartmental model for cobalt (green arrow showing transfer from HRTM–oesophagus slow (Oes S) to the alimentary tract contents) (ICRP 2006, 2015). The subsequent dissolution into blood (systemic pool) from HRTM is indicated with the blue arrow, which is further distributed in organs and tissues in the rest of the body via a systemic model for cobalt (ICRP 2016a). The systemic model of cobalt was reproduced with permission from the ICRP Publication 134, figure 8.1 (ICRP 2016b).

**Figure 2.**
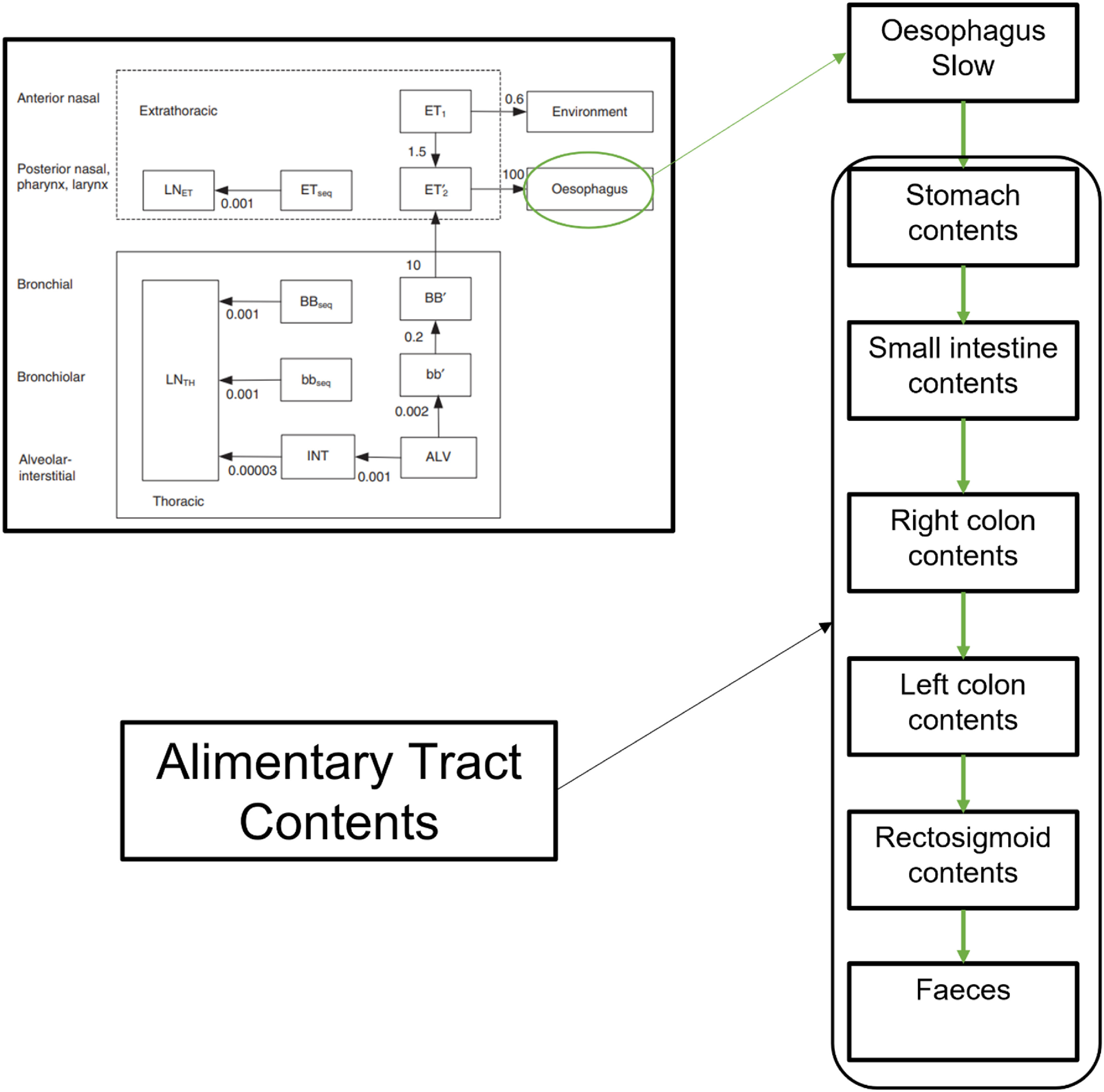
Transfer from HRTM to the alimentary tract contents for cobalt. From the ICRP Publication 100 (ICRP 2006), the human respiratory tract is connected to the digestion system through the slow component of the Oesophagus. The HRTM (top left) was reproduced with permission from the ICRP publication 130, figure 3.4 (ICRP 2015).

**Figure 3.**
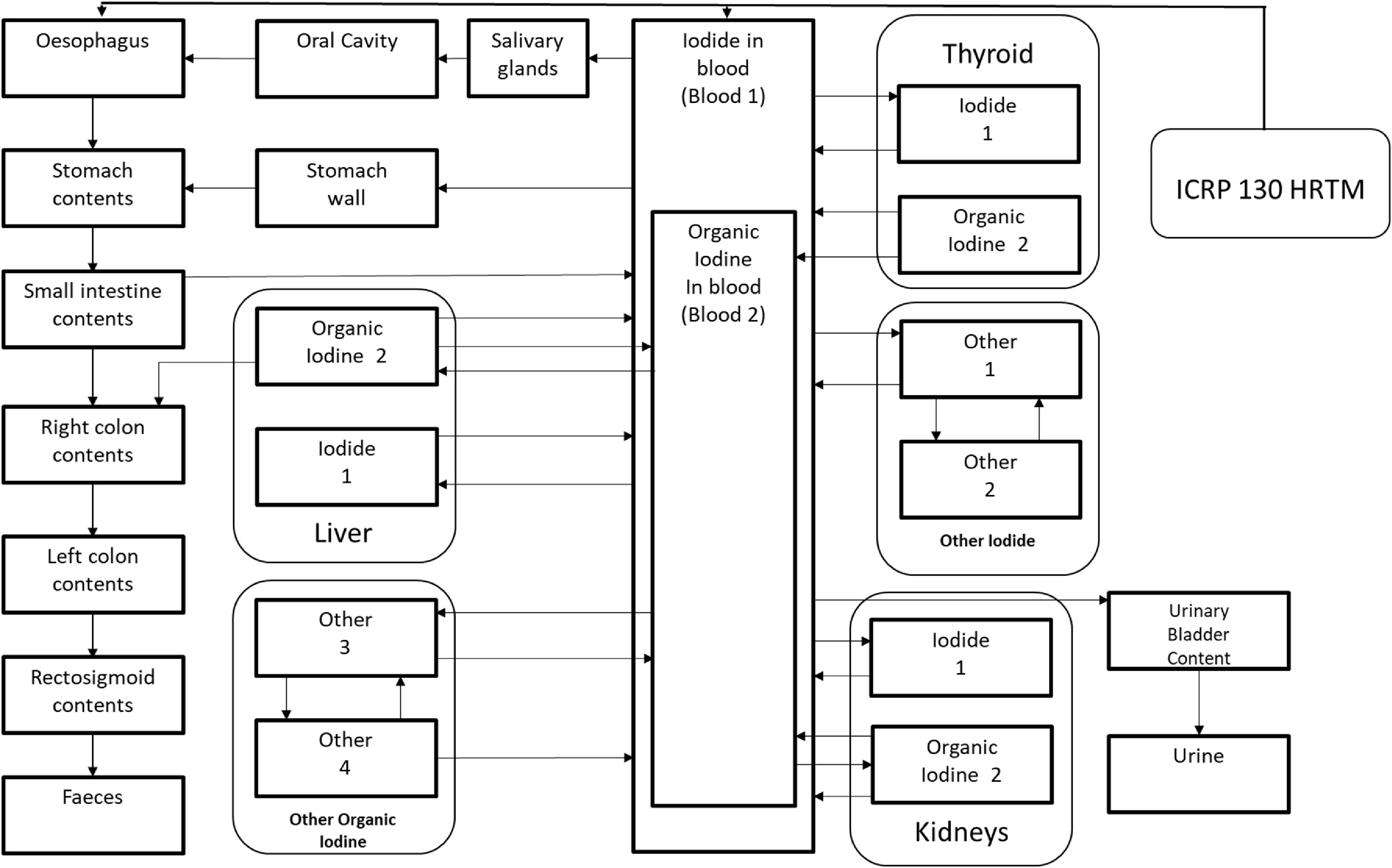
Coupled Inhalation compartmental model for ^131^I (ICRP 2017). Arrows indicate a transfer of iodine from one organ/tissue to another with a designated transfer coefficient in units per day (d^−1^). Similar to the model for cobalt, the respiratory tract is connected to the alimentary tract through the Oesophagus. The systemic model for iodine was reproduced with permission from the ICRP Publication 137, figure 5.2 (ICRP 2017).

The ICRP Publication 130 HRTM is coupled to the radionuclide-specific ^60^Co (ICRP 2016b) and ^131^I (ICRP 2017) systemic models through absorption into blood (ICRP 2015) and the HATM (ICRP 2006), where the complete coupled compartmental model is illustrated in figures 1–3, respectively. In contrast to the systemic models, the HRTM adds complexity to the coupled system of the HRTM and the systemic models through competing clearance modes (mechanical clearance and clearance due to particle dissolution and uptake).

Assuming the clearance classes of Type M for ^60^Co and Type F for ^131^I, respectively, with the particle size of 5 *µ*m, which is the baseline assumption for occupational exposure, Python explicit and implicit ODE solvers and a Python-based matrix exponentiation solving method were used to solve for the coupled system for a unit intake of activity in the human respiratory tract over a 50 year time period. The unit intake of the activity is partitioned into various regions of the human respiratory tract based on the ICRP Publication 130 HRTM for a normal nose breather. These apportionments were read as non-zero initial conditions (fractional deposition) by the ODE-solving methods. The solution of the set of ODEs was evaluated as a fraction of the activity of the radionuclide in each compartment. Discussion of the stability performance for each method was conducted to test the solvers via an adult occupational exposure selected as the reference model (ICRP 2015).

### 2.4. Stiffness and stability

For a coupled linear system of equations with constant coefficients matrix, stiffness can be determined by the relative magnitude of the eigenvalues of the coefficients matrix (Anderson 1983). The relative magnitude is also referred to as the stiffness ratio. The larger the stiffness ratio, the higher the stiffness level and the more restrictive the stability criteria. A large stiffness ratio is evident if eigenvalues of the coefficient matrix are negative or have negative real parts and are widely sparse, posing some difficulties when evaluating the system of ODEs (Anderson 1983, Byrne and Hindmarsh 1987). For each of the coefficient matrices, equation (9) is used to measure the model’s stiffness.

\begin{equation*}{\text{Stiffness Ratio }}\left( {{\text{SR}}} \right) = \,\frac{{\left| {{\lambda _L}} \right|}}{{\left| {{\lambda _S}} \right|}}\, \unicode{x2A7E} \,\frac{{{{\text{max}}_i}\left| {{A_{ii}}} \right|}}{{{{\text{min}}_i}\left| {{A_{ii}}} \right|}}\end{equation*}

where $\left| {{\lambda _L}} \right|$ is the magnitude of the maximum eigenvalue;

$\left| {{\lambda _S}} \right|$ is the magnitude of the minimum eigenvalue;

${\text{ma}}{{\text{x}}_i}\left| {{A_{ii}}} \right|$ is the maximum magnitude value of the diagonal or the loss term of the coefficient matrix; and

${\text{mi}}{{\text{n}}_i}\left| {{A_{ii}}} \right|$ is the minimum magnitude value of the diagonal or the loss term of the coefficient matrix.

Although the stiffness ratio has been a commonly referenced approach for measuring stiffness, it breaks down in the case of real positive eigenvalues, specifically zero eigenvalues resulting in undefine ratio. To obtain useful information on the measure of stiffness for both Type M ^60^Co and Type F ^131^I, zero eigenvalues were discarded, and the stiffness ratio was evaluated with the real negative eigenvalues. The stiffness ratio for ^60^Co and ^131^I were evaluated as $1.03 \times {10^{17}}$ and $6.024 \times {10^{22}}$, respectively. A system with mixed types of eigenvalues (positive and negative) is essentially an unstable system.

Stability, conversely, is a property that ensures that the error associated with the numerical approximation does not grow and thus ensures that the ODE solution does not diverge over time (Yano *et al* 2012). For a detailed stability analysis of the initial value problem posed by the biokinetic models beyond the measure of stiffness, a numerical experiment was performed, and a growth factor known as the numerical amplification factor (G) was employed.

\begin{equation*}{\text{G}} = \,\left| {\frac{{{q_{n + 1}}}}{{{q_n}}}} \right|\, \unicode{x2A7D} \,1\end{equation*}

where ${q_{n + 1}}$ is the numerical solution for the current time step, and ${q_n}$ is the numerical solution for the previous time step. The solution is classified as absolutely stable if, for all time steps, G is less than or equal to 1 over time (Yano *et al* 2012, Patera and Yano 2014).

### 2.5. Computational platform

For this study, a standard personal computer (PC) was utilized as the platform to solve biokinetic problems and to demonstrate the ability to perform such calculations without complex computational resources. The PC is an HP computer that operates on Windows 10. The platform is a 64-bit operating system type with the processor specification as an Intel(R) Core (TM) i7–1065G7 CPU @ 1.30 GHz 1.50 GHz on an 8GB RAM system.

## 3. Results

For an eventual coupling with a multi-level parallel object-oriented framework for advanced parametric analysis, the HRTM biokinetic model was reconstructed and solved in Python for a unit activity of inhaled ^60^Co of Type M and ^131^I of Type F. In this study, initial value problem (IVP) solvers and solving methods were leveraged in Python to numerically and algebraically solve the system of ODEs posed by the biokinetic models and to explore the tolerance levels of the solving methods, hence the choice of moderate and fast clearing radionuclides.

**Figure 4.**
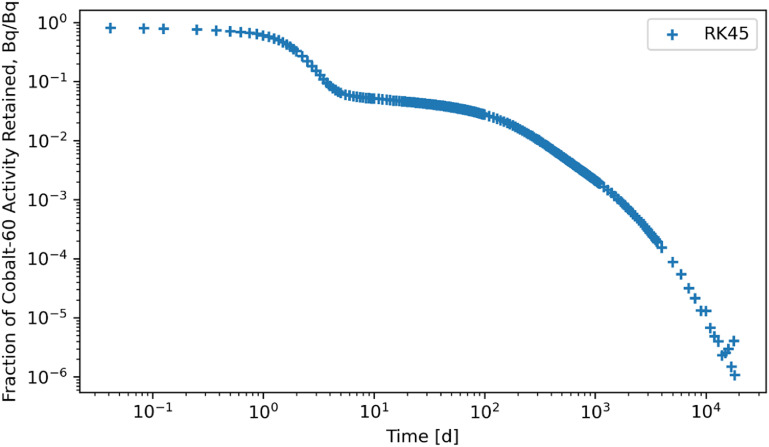
Retention for whole body computed for ^60^Co Type M. The retention solution was obtained with the Python-based explicit Runge–Kutta method of order 5(4)–RK45. This yielded a diverging solution while computationally expensive for the biokinetic solving system and resulted in negative and oscillatory solutions over time.

**Figure 5.**
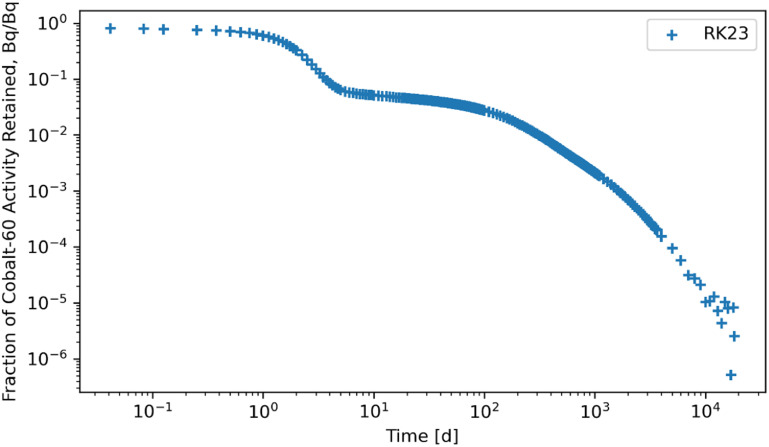
Retention for whole body computed for ^60^Co Type M. The retention solution was obtained with the Python-based explicit Runge–Kutta method of order 3(2)–RK23. This method was computationally expensive for the biokinetic problem set and resulted in negative solutions.

**Figure 6.**
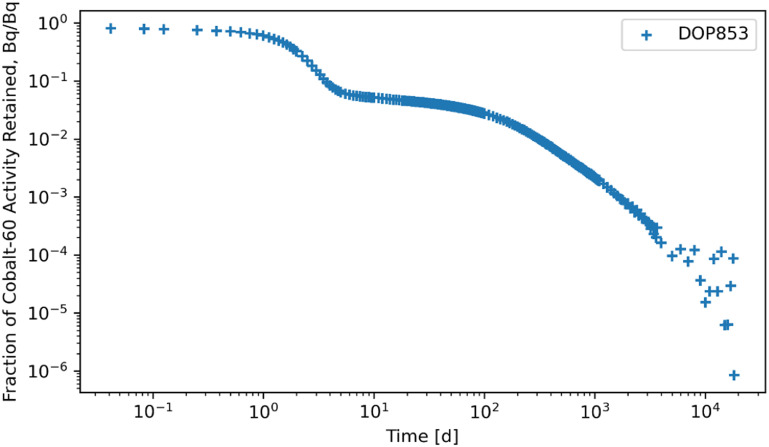
Retention for whole body computed for ^60^Co Type M. The retention solution was obtained with the Python-based explicit Runge–Kutta method of order 8–DOP853. This method was computationally expensive for the biokinetic problem set and resulted in negative and oscillatory solutions.

**Figure 7.**
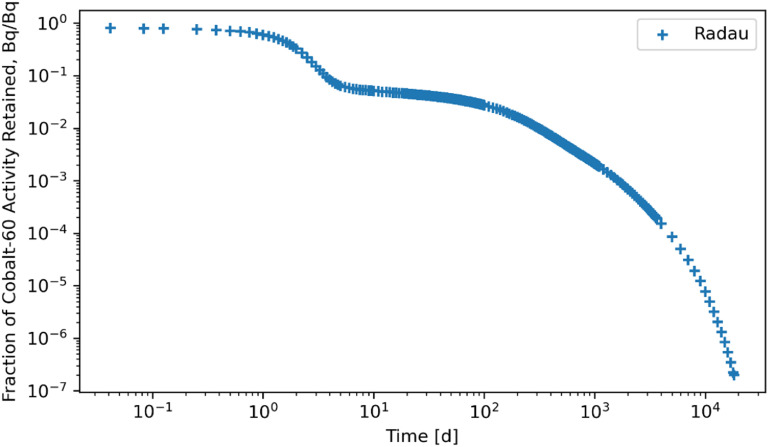
Retention for whole body computed for ^60^Co Type M. The retention solution was obtained with the Python-based implicit Runge–Kutta method of the Radau IIA family of order 5. As a fast calculation for the biokinetic problem set employing default implementation, results yielded some negative solutions due to the large tolerances by defaults (negative solutions are not graphically visible but are present). This is not very evident on the plot but careful looked at the values in the output shows negative solution values.

**Figure 8.**
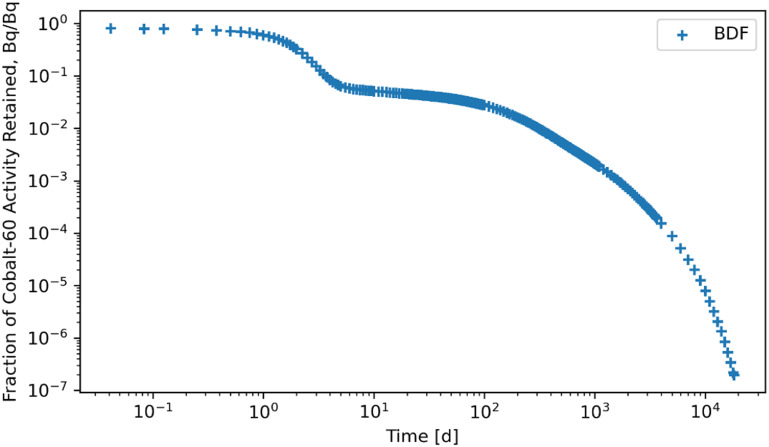
Retention for whole body computed for ^60^Co Type M. The retention solution was obtained with the Python-based implicit multi-step variable-order (1–5) method. As a fast calculation for the biokinetic problem set, results yielded some negative solutions due to the large tolerances by defaults (negative solutions are not graphically visible but are present).

**Figure 9.**
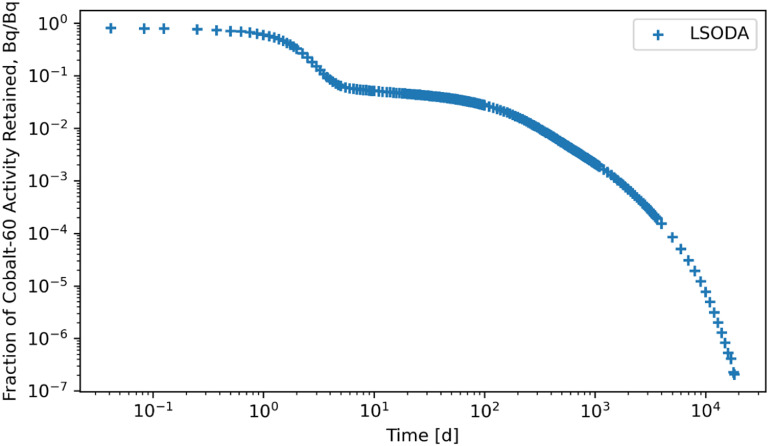
Retention for whole body computed for ^60^Co Type M. The retention solution was obtained with the Python-based adams/BDF method. As a fast calculation for the biokinetic problem set, results yielded negative and oscillatory values in some compartments giving a diverging solution.

Figures 4–9 give the total whole-body retention solutions of the system of coupled ordinary differential equations solved with the following Python solvers: RK45, RK23, DOP853, Radau, BDF, and LSODA for a unit activity of inhaled ^60^Co of Type M, respectively. As shown in figures 4–6, oscillating solutions were observed for RK45, RK23, and DOP853. For Radau, BDF, and LSODA, as shown in figures 7–9, the retention curves for the whole body did not yield oscillatory or divergent solutions.

**Figure 10.**
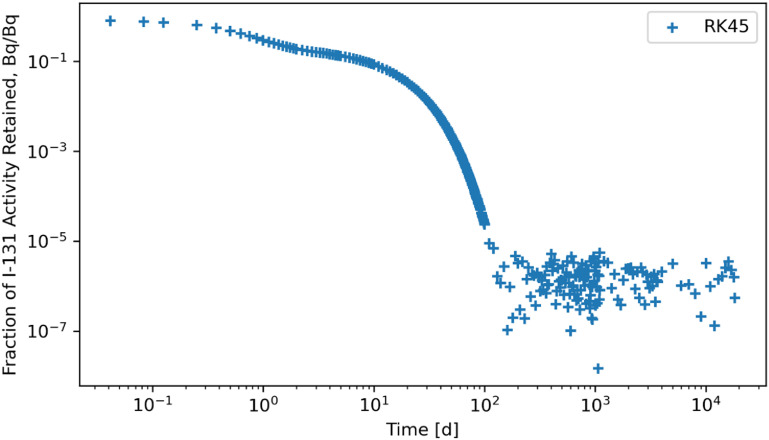
Whole body retention for ^131^I Type F. The retention solution was obtained with the Python-based explicit Runge–Kutta method of order 5(4)–RK45. This method was computationally expensive and resulted in significantly unstable solutions with significant oscillations after ∼ 90 d, where the solution diverges.

**Figure 11.**
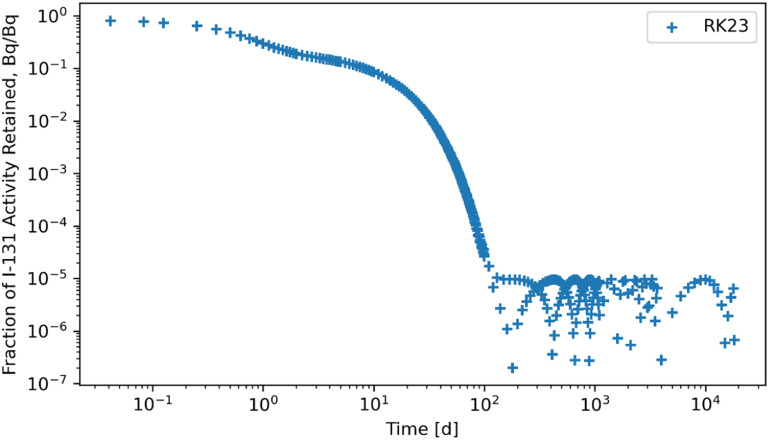
Whole body retention for ^131^I Type F. The retention solution was obtained with the Python-based explicit Runge–Kutta method of order 3(2)—RK23. This method was computationally expensive for the biokinetic problem set and resulted in significantly unstable solutions with significant oscillations after ∼90 d, where the solution diverges.

**Figure 12.**
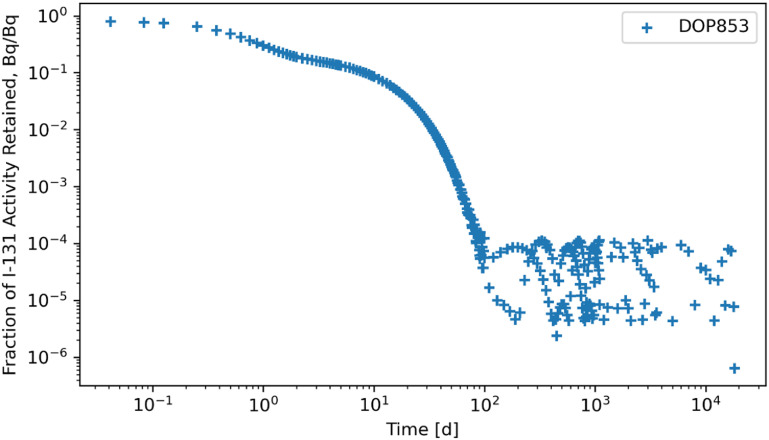
Whole body retention for ^131^I Type F. The retention solution was obtained with the Python-based explicit Runge–Kutta method of order 8–DOP853. This method was computationally expensive for the biokinetic problem set and resulted in significantly unstable solutions with significant oscillations over time, resulting in a diverging solution.

**Figure 13.**
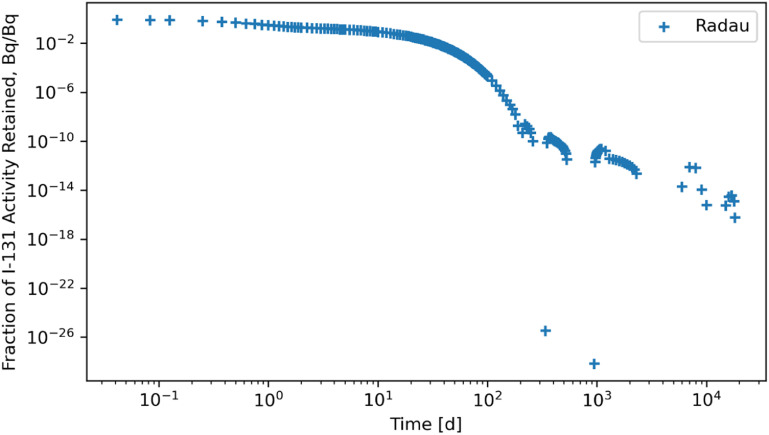
Whole body retention for ^131^I Type F. The retention solution was obtained with the Python-based implicit Runge–Kutta method of the Radau IIA family of order 5. As a fast calculation for the biokinetic problem set, results yielded significantly unstable and oscillatory solutions. Default settings of the solver parameters were used for this case.

**Figure 14.**
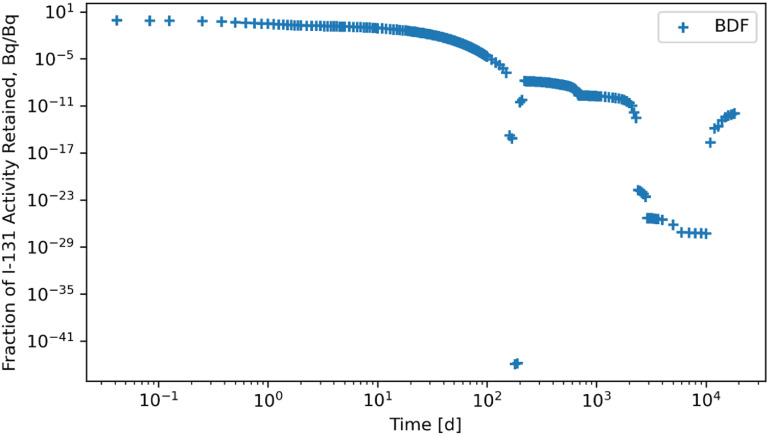
Whole body retention for ^131^I Type F. The retention solution was obtained with the Python-based implicit multi-step variable-order (1–5) method. As a fast calculation for the biokinetic problem set, results yielded significantly unstable and oscillatory solutions. Default settings of the solver parameters were used for this case.

**Figure 15.**
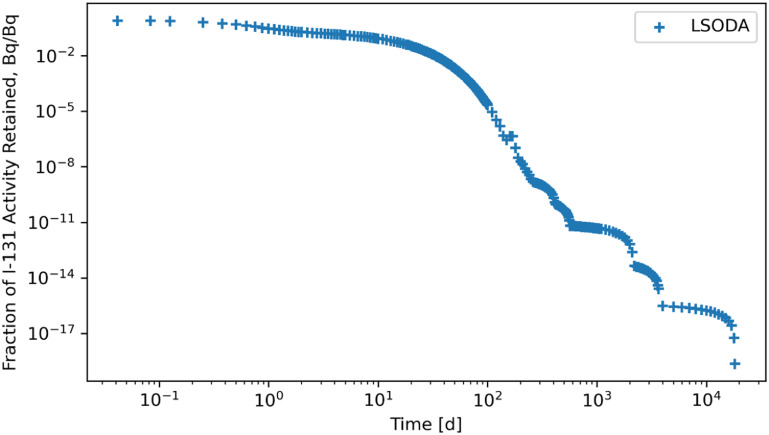
Whole body retention for ^131^I Type F. The retention solution was obtained with the Python-based adams/BDF method. As a fast calculation for the biokinetic problem set, results yielded significantly unstable and oscillatory solutions.

The RK45, RK23, DOP853, Radau, BDF, and LSODA solvers were also subjected to a system of ODEs posed by inhaling ^131^I of Type F and the solutions for whole body retention are reported in figures 10–15. These results demonstrated that all experimented solvers (both implicit and explicit) for the total body activity retained resulted in significant oscillations for the ^131^I scenario. As a result, the optional capabilities for the implicit solvers were further probed by customizing the optional function parameters since the above computations for ^60^Co and ^131^I using these implicit methods implemented the default approach. The choice for specifically adapting the parameters for the implicit methods (Radau and BDF) was based on the recommendation by Python SciPy developers proposing the utility of implicit methods for instances where there are unusually many iterations (divergence) of the solution (Virtanen *et al* 2020). Moreover, explicit solvers utilized several minutes performing many integrations to approximate the solutions, thereby negating further exploration. Customization in this study utilized the optional capability of the implicit solvers to regulate the local error in computing the solution, as well as optimizing the computational time, as detailed in the methodology. Comparable outcomes for these solvers were observed for both the human respiratory tract and whole-body retention, depicted in the graphical data for whole-body retention as a representation of the global stability effect.

**Figure 16.**
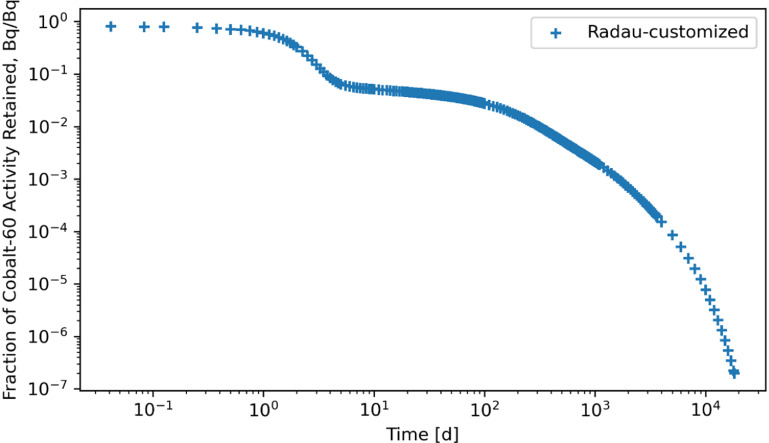
Whole body retention for ^60^Co Type M. The retention solution was obtained with the Python-based solvers implicit Runge–Kutta method of the Radau IIA family of order 5. As a fast calculation for the biokinetic problem set, results yielded stable solutions. This solution depicts the instance of enhancing the local error control for Radau by reducing the relative and absolute tolerances.

**Figure 17.**
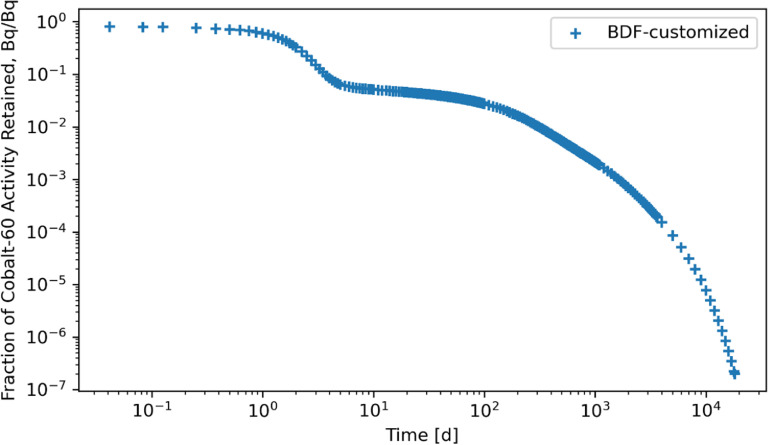
Whole body retention for ^60^Co Type M. The retention solution was obtained with the Python-based implicit multi-step variable-order (1–5) method. As a fast calculation for the biokinetic problem set, results yielded few oscillations in a few compartments between outputting exactly 0 and values close to 0 (10^−45^), which can essentially be considered zero. This solution depicts the instance of enhancing the local error control for BDF by reducing the relative and absolute tolerances.

**Figure 18.**
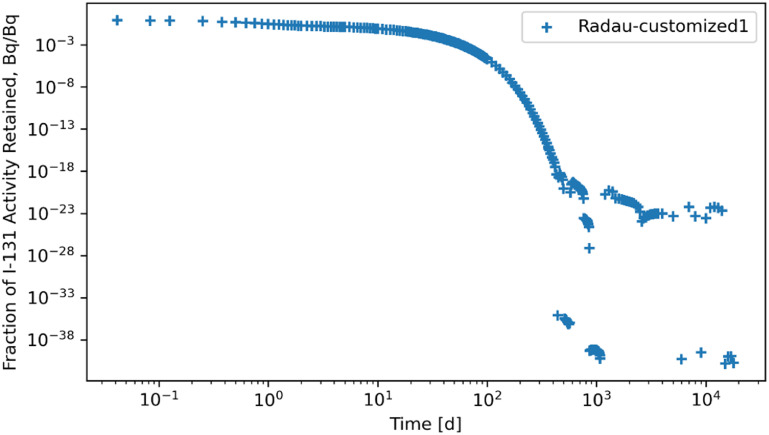
Whole body retention for ^131^I Type F. The retention solution was obtained with the Python-based implicit Runge–Kutta method of the Radau IIA family of order 5. As a fast calculation for the biokinetic problem set, results yielded significantly unstable solutions. This solution depicts the instance for enhancing the local error control for Radau by reducing the relative and absolute tolerances.

**Figure 19.**
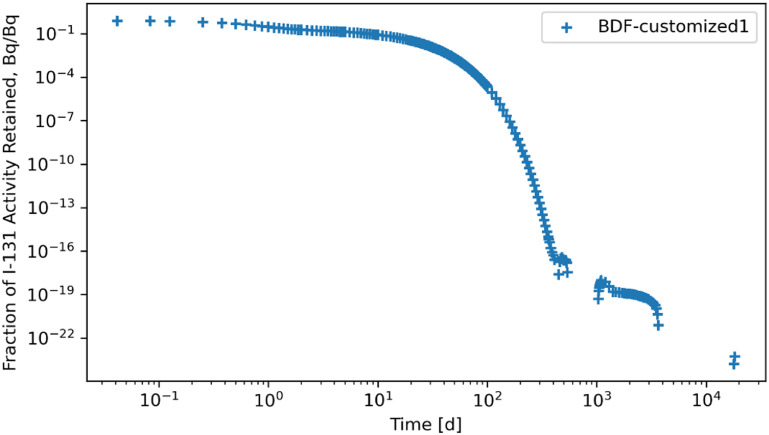
Whole body retention for ^131^I Type F. The retention solution was obtained with the Python-based implicit multi-step variable-order (1–5) method. As a fast calculation for the biokinetic problem set, results yielded significantly unstable solutions. This solution depicts the instance for enhancing the local error control for BDF by reducing the relative and absolute tolerances.

**Figure 20.**
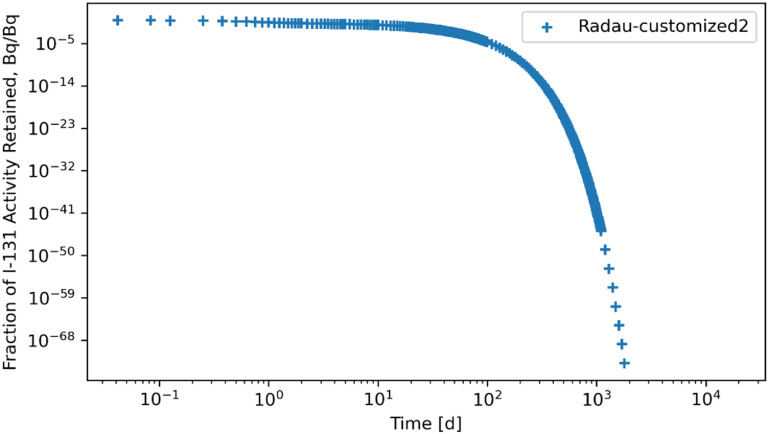
Whole body retention for ^131^I Type F. The retention solution was obtained with the Python-based Runge–Kutta method of the Radau IIA family of order 5. As a fast calculation for the biokinetic problem set, results yielded stable solutions. A stable solution was obtained by restricting the step size to a maximum of 0.2 in addition to local error control via relative and absolute tolerances.

**Figure 21.**
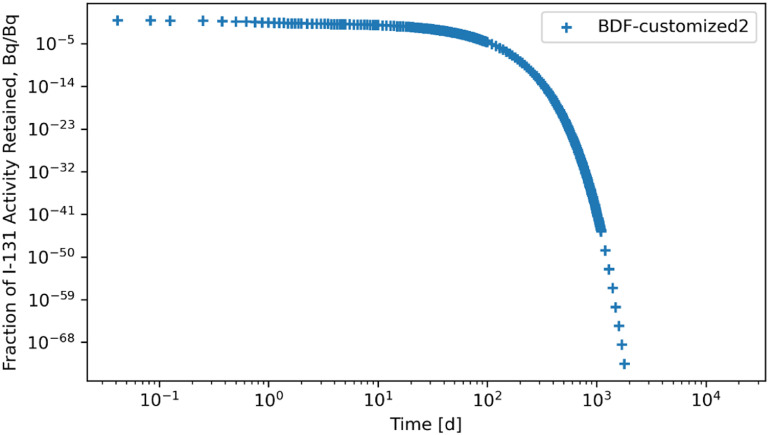
Whole body retention for ^131^I Type F. The retention solution was obtained with the Python-based implicit multi-step variable-order (1–5) method. As a fast calculation for the biokinetic problem set, results yielded stable solutions. A stable solution was obtained by restricting the step size to a maximum of 0.2 in addition to local error control via relative and absolute tolerances.

Retention results are given in figures 16–21, from which solutions were obtained utilizing the local error monitoring capabilities and controlling the step size for the numerical integration of Radau and BDF for ^60^Co of Type M and ^131^I of Type F, respectively.

**Figure 22.**
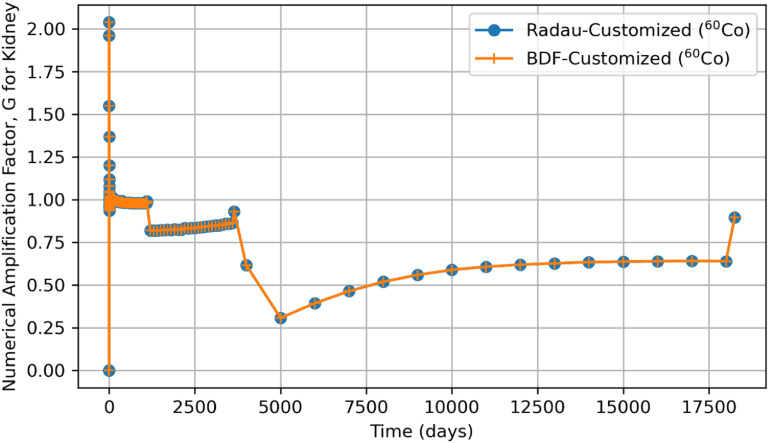
Stability analysis of the Python-based solvers (Radau and BDF) using numerical amplification factor, G over 50-year time span for Kidney retention solutions of ^60^Co.

**Figure 23.**
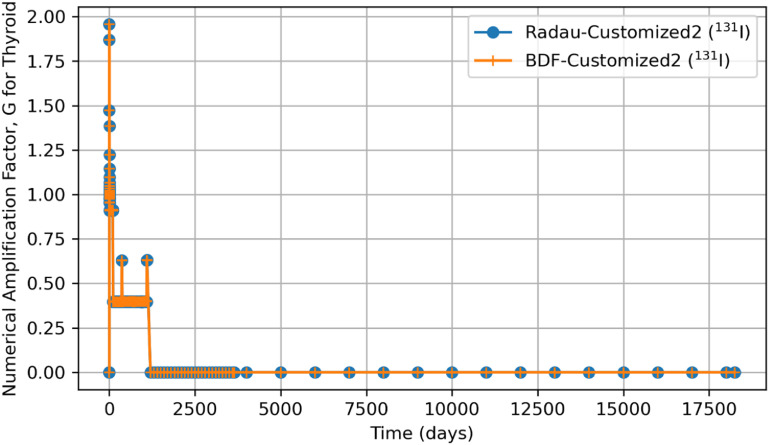
Stability analysis of the Python-based solvers (Radau and BDF) using numerical amplification factor, G over 50 year time span for computed activity retained in the Thyroid of ^131^I.

Tables 2 and 3 report the calculation time for each of the solvers used and indicate solvers that resulted in oscillatory or divergent solutions in specific compartments of the biokinetic model problem. It is important to note that despite the apparent oscillations observed in some of the solutions for the solvers for the ^131^I scenario, oscillations in ^60^Co were negligible. However, these non-oscillatory solutions do not necessarily indicate stability. Tables 4–7 provide the summary results from the stability analysis of the solvers quantitatively for ^60^Co of Type M and ^131^I of Type F, respectively, showing the maximum G, as defined in equation (10). Although the maximum G values of Radau-Customized and BDF-Customized for ^60^Co, as well as of Radau-Customized2 and BDF-Customized2 for ^131^I, were slightly greater than 1, a trend was observed over time which further examined the stability metric in this problem framework. Figures 22 and 23 provide an illustration of this trend for *Kidneys* (^60^Co) and *Thyroid* (^131^I).

**Table 2.** Solvers with their calculation time on a standard Windows 10 OS computer for ^60^Co biokinetic model.

Method	Solver	Solving time (s)	Specific compartment divergence
Explicit	RK45	1314.00	High
	RK23	1870.32	High
	DOP853	1730.32	High
Implicit	Radau	0.10	High
	Radau-Customized	2.54	None
	BDF	0.10	High
	BDF-Customized	1.00	Moderate
Universal choice	LSODA	0.04	High

**Table 3.** Solvers with their calculation time on a standard Windows 10 OS computer for ^131^I biokinetic model.

Method	Solver	Solving time [s]	Specific compartment divergence
Explicit	RK45	1462.50	High
	RK23	1068.41	High
	DOP853	2669.40	High
Implicit	Radau	0.13	High
	Radau-Customized1	2.75	High
	Radau-Customized2	64.34	None
	BDF	0.15	High
	BDF-Customized1	0.50	High
	BDF-Customized2	16.00	None
Universal choice	LSODA	0.03	High

**Table 4.** Stability analysis of the Python-based solvers using numerical amplification factor, G for ^60^Co summarizing the analysis by providing the maximum G for whole body retention.

Method	Solver	Maximum numerical amplification factor, G (Whole body)
Explicit	RK45	2.73 × 10^0^
	RK23	1.59 × 10^1^
	DOP853	4.77 × 10^0^
Implicit	Radau	9.94 × 10^−1^
	Radau-Customized	9.94 × 10^−1^
	BDF	9.94 × 10^−1^
	BDF-Customized	9.94 × 10^−1^
Universal choice	LSODA	9.94 × 10^−1^

**Table 5.** Stability analysis of the Python-based solvers using numerical amplification factor, G for ^60^Co summarizing the analysis by providing the maximum G for all relevant regions of the biokinetic model.

Method	Solver	Maximum G (Respiratory tract)	Maximum G (Oesophagus)	Maximum G (Blood)	Maximum G (Liver)	Maximum G (Alimentary tract contents & walls)	Maximum G (Bones)	Maximum G (Other soft tissues)	Maximum G (Kidneys)	Maximum G (UBC)
Explicit	RK45	9.94 × 10^−1^	1.83 × 10^2^	1.48 × 10^0^	2.04 × 10^0^	5.42 × 10^2^	2.05 × 10^0^	2.05 × 10^0^	2.04 × 10^0^	1.67 × 10^0^
	RK23	9.94 × 10^−1^	7.23 × 10^1^	1.48 × 10^0^	2.04 × 10^0^	2.41 × 10^2^	1.75 × 10^−3^	2.05 × 10^0^	2.04 × 10^0^	1.67 × 10^0^
	DOP853	9.94 × 10^−1^	2.52 × 10^1^	2.63 × 10^0^	2.04 × 10^0^	5.27 × 10^2^	2.05 × 10^0^	2.05 × 10^0^	2.04 × 10^0^	1.91 × 10^0^
Implicit	Radau	2.25 × 10^0^	1.19 × 10^1^	1.48 × 10^0^	2.04 × 10^0^	1.18 × 10^0^	2.05 × 10^0^	2.05 × 10^0^	2.04 × 10^0^	1.67 × 10^0^
	Radau-Customized	9.94 × 10^−1^	9.96 × 10^−1^	1.48 × 10^0^	2.04 × 10^0^	1.18 × 10^0^	2.05 × 10^0^	2.05 × 10^0^	2.04 × 10^0^	1.67 × 10^0^
	BDF	2.40 × 10^1^	8.18 × 10^0^	1.48 × 10^0^	4.21 × 10^0^	1.18 × 10^0^	2.05 × 10^0^	9.24 × 10^0^	8.60 × 10^0^	1.67 × 10^0^
	BDF-Customized	9.94 × 10^−1^	9.95 × 10^−1^	1.48 × 10^0^	2.04 × 10^0^	1.18 × 10^0^	2.05 × 10^0^	2.05 × 10^0^	2.04 × 10^0^	1.67 × 10^0^
Universal choice	LSODA	2.02 × 10^17^	2.02 × 10^1^	1.48 × 10^0^	3.39 × 10^0^	1.18 × 10^0^	2.05 × 10^0^	2.55 × 10^0^	7.45 × 10^0^	1.67 × 10^0^

**Table 6.** Stability analysis of the Python-based solvers using numerical amplification factor, G for ^131^I summarizing the analysis by providing the maximum G for whole body retention.

Method	Solver	Maximum numerical amplification factor, G (whole body)
Explicit	RK45	2.27 × 10^2^
	RK23	4.84 × 10^1^
	DOP853	2.26 × 10^1^
Implicit	Radau	2.85 × 10^16^
	Radau-Customized1	1.11 × 10^18^
	Radau-Customized2	9.78 × 10^−1^
	BDF	2.00 × 10^33^
	BDF-Customized1	7.52 × 10^0^
	BDF-Customized2	9.78 × 10^−1^
Universal choice	LSODA	1.61 × 10^0^

**Table 7.** Stability analysis of the Python-based solvers using numerical amplification factor, G for ^131^I summarizing the analysis by providing the maximum G for all relevant regions of the biokinetic model.

Method	Solver	Maximum G (Respiratory tract)	Maximum G (Oesophagus)	Maximum G (Oral cavity)	Maximum G (Salivary gland)	Maximum G (Blood)	Maximum G (Thyroid)
Explicit	RK45	9.13 × 10^−1^	1.02 × 10^2^	2.27 × 10^2^	1.18 × 10^1^	1.85 × 10^10^	2.27 × 10^2^
	RK23	9.13 × 10^−1^	8.06 × 10^1^	3.50 × 10^1^	4.34 × 10^1^	5.98 × 10^10^	6.07 × 10^2^
	DOP853	9.13 × 10^−1^	2.40 × 10^1^	1.56 × 10^1^	1.94 × 10^1^	3.79 × 10^10^	4.85 × 10^2^
Implicit	Radau	1.25 × 10^27^	3.82 × 10^1^	3.82 × 10^1^	3.83 × 10^1^	3.83 × 10^1^	3.82 × 10^1^
	Radau-Customized1	1.73 × 10^3^	1.36 × 10^1^	1.36 × 10^1^	1.36 × 10^1^	1.52 × 10^1^	1.68 × 10^1^
	Radau-Customized2	9.13 × 10^−1^	1.02 × 10^0^	1.13 × 10^0^	1.12 × 10^0^	1.03 × 10^0^	1.96 × 10^0^
	BDF	3.53 × 10^22^	5.49 × 10^1^	5.50 × 10^1^	5.45 × 10^1^	4.46 × 10^1^	3.01 × 10^4^
	BDF-Customized1	9.13 × 10^−1^	7.52 × 10^0^	7.52 × 10^0^	7.52 × 10^0^	7.52 × 10^0^	7.52 × 10^0^
	BDF-Customized2	9.13 × 10^−1^	1.02 × 10^0^	1.13 × 10^0^	1.12 × 10^0^	1.03 × 10^0^	1.96 × 10^0^
Universal choice	LSODA	1.04 × 10^8^	2.77 × 10^0^	2.79 × 10^0^	2.75 × 10^0^	4.04 × 10^0^	3.40 × 10^−1^

Method	Solver	Maximum G (Liver)	Maximum G (Alimentary tract contents & walls)	Maximum G (Other soft tissues)	Maximum G (Kidneys)	Maximum G(UBC)
Explicit	RK45	1.11 × 10^10^	8.26 × 10^2^	2.98 × 10^2^	1.08 × 10^11^	1.15 × 10^1^
	RK23	1.31 × 10^10^	2.69 × 10^3^	1.61 × 10^3^	1.28 × 10^11^	2.87 × 10^1^
	DOP853	2.66 × 10^10^	1.71 × 10^3^	1.28 × 10^3^	2.58 × 10^11^	1.93 × 10^1^
Implicit	Radau	3.82 × 10^1^	3.83 × 10^1^	3.83 × 10^1^	3.84 × 10^1^	3.82 × 10^1^
	Radau-Customized1	1.79 × 10^1^	1.39 × 10^1^	1.47 × 10^1^	1.48 × 10^1^	1.36 × 10^1^
	Radau-Customized2	1.04 × 10^0^	1.01 × 10^0^	1.05 × 10^0^	1.03 × 10^0^	1.62 × 10^0^
	BDF	2.44 × 10^2^	9.51 × 10^1^	4.99 × 10^1^	4.90 × 10^1^	1.76 × 10^2^
	BDF-Customized1	7.52 × 10^0^	7.51 × 10^0^	7.51 × 10^0^	7.51 × 10^0^	7.52 × 10^0^
	BDF-Customized2	1.04 × 10^0^	1.01 × 10^0^	1.05 × 10^0^	1.03 × 10^0^	1.62 × 10^0^
Universalchoice	LSODA	4.07 × 10^0^	2.58 × 10^0^	5.00 × 10^0^	4.08 × 10^0^	1.62 × 10^0^

**Figure 24.**
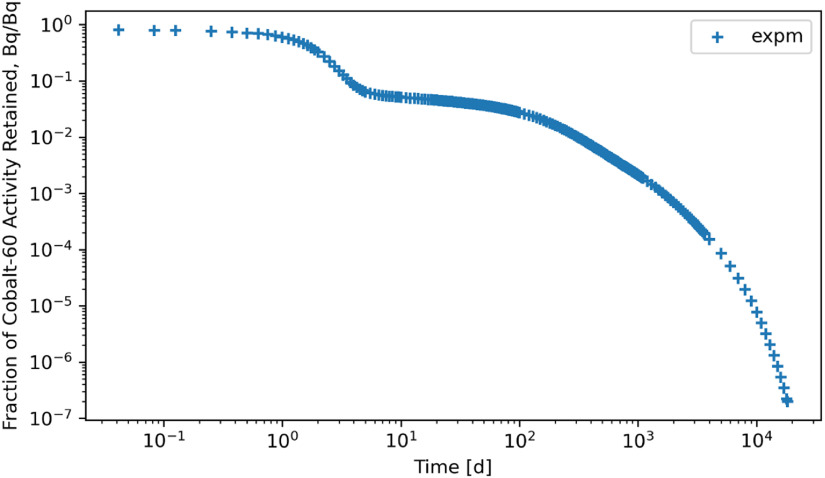
Whole body retention computed using Python-based matrix exponential method for ^60^Co Type M. The result depicts stable solutions with a fast calculation time of approximately 0.4 s.

**Figure 25.**
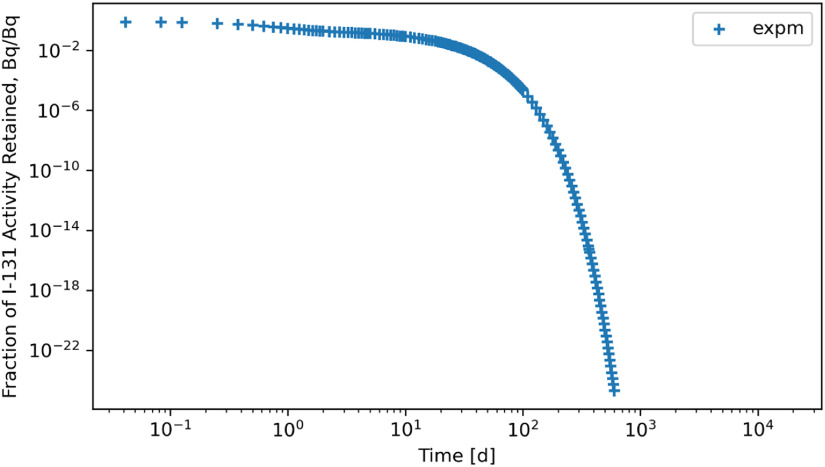
Whole body retention computed using Python-based matrix exponential method for ^131^I Type F. The result depicts stable solutions for the highly stiff system with a fast calculation time of approximately 0.4 s.

**Figure 26.**
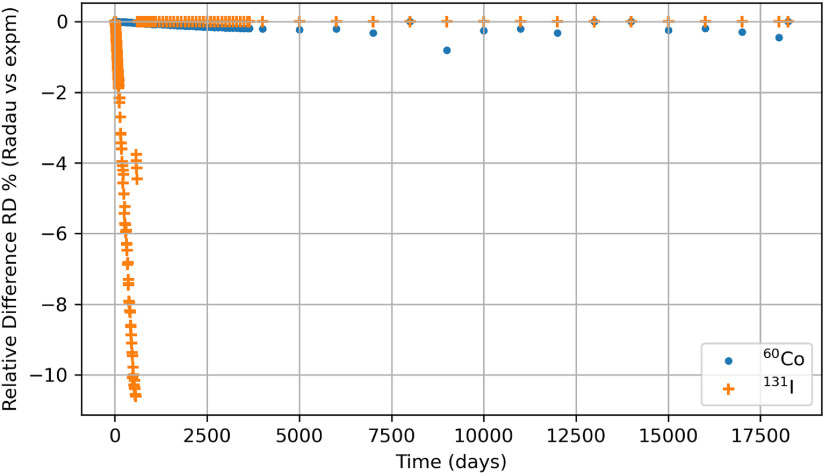
The percent relative difference (RD %) for whole body retention solution between Python-based *expm* and Radau (Customized for ^60^Co and Customized 2 for ^131^I) for a 50 year time period.

**Figure 27.**
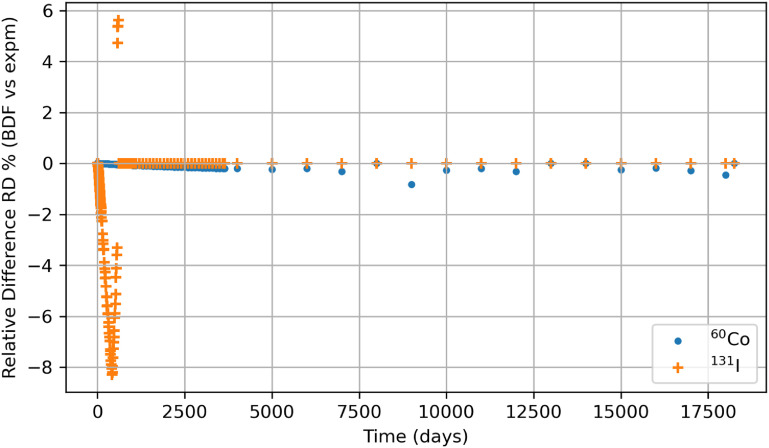
The percent relative difference (RD %) for whole body retention solution between Python-based *expm* and BDF (Customized for ^60^Co and Customized 2 for ^131^I) for a 50 year time period.

Stiff problems mostly favor solution methods based on eigenvalue methodologies and, thus, algebraic methods. This study made a conscious effort to compute the biokinetic solutions for the radionuclides using a linear algebraic method (*expm*) in Python, which employs Padé approximation for which figures 24 and 25 represent the retention of whole-body radioactivity burden for ^60^Co Type M and ^131^I Type F, respectively. For verification purposes, the retention resulting from this method was then compared with the solutions of the customized Python IVP implicit solvers by finding the percent relative difference between *expm* and Radau, ((Radau-*expm*/*expm*) $ \times $ 100) and *expm* and BDF ((BDF-*expm*/*expm*) $ \times $ 100) for ^60^Co of Type M and ^131^I of Type F. The results for the relative difference are shown in figures 26 and 27.

As indicated in the methodology, the inhalation scenarios for ^60^Co of Type M and for ^131^I of Type F were derived from the ICRP OIR series of publications (ICRP 2015, 2016b, 2017). As a result, the biokinetic retention solutions from OIR Electronic Annex Distribution Set were compared with the solutions obtained with the solvers/solving methods. The percent relative difference (RD %) for total body retention are reported in tables 8 and 9.

**Table 8.** The percent relative difference (RD %) for total body retention between ICRP OIR Electronic annex distribution set and the Python solvers/solving methods investigated for ^60^Co.

Solvers/Methods	Relative difference (RD%)–Total body activity retained for 5 *µ*m
RK45 versus ICRP OIR	−1.71 × 10^0^ ⩽ RD ⩽ 1.69 × 10^3^
RK23 versus ICRP OIR	−1.62 ×10^0^ ⩽ RD ⩽ 3.50 × 10^3^
DOP853 versus ICRP OIR	−1.23 × 10^0^ ⩽ RD ⩽ 3.81 × 10^4^
LSODA versus ICRP OIR	−3.93 × 10^0^ ⩽ RD ⩽ 1.66 × 10^1^
BDF versus ICRP OIR	−3.63 × 10^0^ ⩽ RD ⩽ 1.09 × 10^0^
BDF_Customized versus ICRP OIR	−2.12 × 10^0^ ⩽ RD ⩽ 2.00 × 10^−3^
Radau versus ICRP OIR	−2.20 × 10^0^ ⩽ RD ⩽ 2.00 × 10^−3^
Radau_Customized versus ICRP OIR	−2.12 × 10^0^ ⩽ RD ⩽ 2.00 × 10^−3^
expm versus ICRP OIR	−1.73 × 10^0^ ⩽ RD ⩽ 2.00 × 10^−3^

**Table 9.** The percent relative difference (RD %) for total body retention between ICRP OIR Electronic annex distribution set and the Python solvers/solving methods investigated for ^131^I.

Solvers/Methods	Relative difference (RD%)–Activity retained (Total body) for 5 *µ*m
RK45 versus ICRP OIR	−1.54 × 10^0^ ⩽ RD ⩽ 1.24 × 10^8^
RK23 versus ICRP OIR	−1.10 × 10^0^ ⩽ RD ⩽ 4.10 × 10^8^
DOP853 versus ICRP OIR	−6.70 × 10^−1^ ⩽ RD ⩽ 3.98 × 10^9^
LSODA versus ICRP OIR	−1.37 × 10^1^ ⩽ RD ⩽ 9.69 × 10^4^
BDF versus ICRP OIR	−1.00 × 10^2^ ⩽ RD ⩽ 9.34 × 10^5^
BDF_Customized1 versus ICRP OIR	−5.84 × 10^0^ ⩽ RD ⩽ 3.40 × 10^−2^
BDF_Customized2 versus ICRP OIR	−5.84 × 10^0^ ⩽ RD ⩽ 3.40 × 10^−2^
Radau versus ICRP OIR	−1.00 × 10^2^ ⩽ RD ⩽ 2.08 × 10^3^
Radau_Customized1 versus ICRP OIR	−5.84 × 10^0^ ⩽ RD ⩽ 3.40 × 10^−2^
Radau_Customized2 versus ICRP OIR	−5.84 × 10^0^ ⩽ RD ⩽ 3.40 × 10^−2^
expm versus ICRP OIR	−3.00 × 10^−1^ ⩽ RD ⩽ 1.90 × 10^−1^

## 4. Discussion

Several ODE solvers and solving methods in Python were employed for solving the biokinetic models from inhaled ^60^Co of Type M and ^131^I Type F. For the total body retention of inhaled ^60^Co, the examined solvers (figures 4–9) resulted in an unusual iteration except for Radau, BDF, and LSODA, which were not visually observable in the figures. However, careful analysis of the compartmental activity, through a compartment-by-compartment analysis, demonstrated oscillatory solutions in some of the compartments for the solvers except the customized (local error control) Radau solver (table 2), where customized BDF implemented with the same customization of error tolerance as Radau. For ^131^I, solutions with both explicit and implicit solvers in their general form resulted in unstable and oscillatory solutions, as shown in figures 10–15. The relative and absolute tolerances (*rtol* and *atol*) were reduced to enable local error control for accuracy, and the activity retained in each compartment was computed and plotted for the whole body for ^60^Co and ^131^I in figures 16–21, respectively. However, as shown in the plots of figures 18 and 19, there were significant oscillations in the solutions for both Radau and BDF. For ^131^I, the relative and absolute tolerance chosen saturated at 10^−15^ and 10^−12^, respectively, without any significant effect due to the highly stiff nature of the ^131^I coupled HRTM and systemic biokinetics. The resolution implemented entailed restricting the maximum time points to have an upper bound on the error. Figures 20 and 21 depict non-oscillatory (smooth) solutions. The customization confirms the importance of the optional function parameters of the Python solvers where needed and the need to pay particular attention to their usage.

Additionally, as computational time is essential to the development of modeling tools and, thus, internal dosimetry tools, the calculation time of the solvers were tracked, which is reported in tables 2 and 3 for ^60^Co and ^131^I, respectively, indicating that the implicit solvers and a versatile choice (LSODA) were computationally efficient for the biokinetic model problem. These calculation times were system-specific and should not be considered absolute. However, the magnitude of solving speed capabilities for each solver was maintained, regardless of the computer-based system in use. In contrast, explicit solvers, such as RK45, RK23, and DOP853, took approximately 17–35 min to solve the biokinetic model problems, for which the longest time spent achieving a solution was for the case of the coupled ^131^I HRTM and systemic biokinetic model. Thus, to achieve numerically optimal solutions with limited accuracy for stiff problems, explicit solvers performed many integrations with very small step sizes, which can sometimes be considered unacceptable. Also, color-coded cells under ‘specific compartment divergence’ in tables 2 and 3 summarize the solvers that resulted in some level of instability (or oscillatory solutions) and those that were stable in solving the inhalation modeling problems.

### 4.1. Stability evaluation

Quantitatively, a growth factor named the numerical amplification factor, G, was employed for the stability criteria of the numerical solution for the solvers. As shown in table 4, the three explicit solvers, RK45, RK23, and DOP853, resulted in a maximum G factor greater than 1 when tracked over time. The maximum G factor obtained for Radau, Radau-Customized, BDF, BDF-Customized, and LSODA was less than 1 for whole-body retention. A summary of maximum G values reported in table 5 shows that global stability in the total body activity burden does not necessarily entail stability in all cases. From table 5, Radau, Radau-Customized, BDF, BDF-Customized, and LSODA all resulted in maximum G values greater than 1 over time in some compartments with LSODA as the highest (2.02 × 10^17^ in the respiratory tract). For ^131^I, stability analysis for whole body (table 6) generally indicated significant instability with all the solvers (G greater than 1) except for Radau-Customized2 and BDF-Customized2, whose maximum G values were less than 1. Table 7 also provides the maximum G values for all the organs/compartments considered for the biokinetic modeling for ^131^I. Table 7 demonstrates that most compartments under study resulted in G values greater than 1 except for Radau-Customized2 and BDF-Customized2, where, in most cases, maximum G values reported were slightly higher than 1.

It is essential to further emphasize that even though some compartments presented maximum G values that are slightly greater than 1 (as noted for Radau-Customized and BDF-Customized in table 5 and for Radau-Customized2 and BDF-Customized2 in table 7), the stability involves consideration of evolution as a function of time. Thus, the solution is termed stable if the G value, over time, is less than or equal to 1. Additionally, the transient dynamics of the customized solvers in figure 22 (Radau-Customized and BDF-Customized) and figure 23 (Radau-Customized2 and BDF-Customized2) may be associated with factors such as, but not limited to, adaptation of the solver’s time steps, initial conditions, and interaction between compartments. In this study, the transient behavior of the G value for the individual tissues and organs of the customized solvers may be due to the inter-compartmental exchange of material within the body. This is in contrast with the whole-body retention, which would necessarily have G values bounded to values less than 1 due to material losses such as radioactive decay and excretion. However, very large G values over time signify instability. For these instances, a noteworthy trend was that although the growth factor grew to a value greater than 1 (worst case for Radau-Customized and BDF-Customized in table 5 and for Radau-Customized2 and BDF-Customized2 in table 7 has G as 2.05) initially, G rapidly decays to values less than or equal to 1 over time. This trend suggests that the solution is stable, and the uncertainties are effectively reduced in the context of numerical methods. Figures 22 and 23 specifically illustrate the initial growth of the G factor, but eventually resulted in stability. These results confirm that not all implicit solvers, in their general form and solving methods, are suitable for all stiff problems. As observed when using LSODA, although it is computationally efficient, LSODA resulted in significantly unstable solutions in most cases. The unusual instability observed in most cases for LSODA can be associated with the stiffness detection methodology implemented in the algorithm. Ultimately, LSODA solves the ODEs by automatically switching between non-stiff and stiff-solving methods depending on the problem encountered. However, the realization in some cases that implicit solutions will proceed with fast calculations makes the evaluation biased, necessitating altering the step size and ultimately affecting the internal error estimates.

Although exploration of customized implicit methods, such as the Radau and BDF methods, suggested good performance for stiff problems in Python, further investigating a linear algebraic demonstrated its capabilities without the need of explicitly modifying the local error or critically implementing the optional function parameters based on the stiffness level of the system under study (figures 24 and 25). As an exact solution, the algebraic solution (matrix exponential) achieved a calculation time of approximately 0.411 s for ^60^Co and ^131^I, which was then compared to the customized Radau and BDF solvers as given in figures 26 and 27. Results given in figure 26 demonstrate that the numerical implicit Radau solver underestimated the Python-based *expm* with a maximum relative difference of approximately 0.81% and 10.60% for ^60^Co and ^131^I, respectively.

The maximum relative difference observed is attributed to numerical precision and the variance introduced by the notion of relative difference for very small solution values. Additionally, the implicit Radau solver (Radau IIA methods) utilizes sub-diagonal Padé approximation to determine the method’s stability. At the same time, *expm* in Python explicitly implements a new scaling and squaring algorithm for matrix exponentiation with improved accuracy. The results in figure 27 give comparable relative percent differences for ^60^Co in the case of customized BDF; however, for ^131^I, BDF underestimated *expm* with a maximum relative difference of 8.20% but with an overestimation of approximately 5.60% at ∼600 d post-exposure. The differences can be attributed to numerical precision and the method of integration used by BDF for evaluation.

Furthermore, some ODE solving platforms like MATLAB recommended not using maximum step for accuracy and, if used, only as a last resort. The results produced in this work for the utility of maximum step size were compared with the matrix exponentiation approach with relatively good agreement. Overall, the similarity between the solutions for Radau and BDF confirmed the recommendation by SciPy to use Radau and BDF for stiff problems. The methods implemented in these two solvers dictate the accuracy (the nearness to the true solution) of the numerical solution based on the level of stiffness and on which optional function parameters are utilized or adapted. In effect, the differences observed between the implicit methods and *expm*, in addition to numerical precision, could be associated with the order evaluation utilized by the numerical methods.

### 4.2. Comparison with the ICRP OIR electronic annex distribution set

The Python-based biokinetic solutions were compared with electronically available biokinetic data (the ICRP OIR Electronic Annex Distribution Set)–reported in tables 8 and 9. As expected from the instability observed in the instances of the explicit solvers and LSODA, significant percent differences, in general, were observed when compared with the ICRP OIR electronic data. However, with stable solutions (a good G factor and non-oscillatory solution), the customized implicit methods and the algebraic method (*expm*) outlined good agreement with the ICRP OIR Electronic Data predictions as depicted in tables 8 and 9 for ^60^Co and ^131^I, respectively.

## 5. Conclusion

The capabilities of solving a complex system of coupled ODEs for biokinetic modeling, using solvers in Python, were explored, where implicit methods demonstrated practical applicability for both moderate and fast clearing radionuclides through a stability analysis. Characteristic tolerance levels were explored for the constituent in Python by being subjected to rapidly and slowly varying coupled ODE systems. The outcome of the implicit solvers (Radau and BDF) with the stable solutions was then compared to the solution obtained with a straightforward (albeit complex) algebraic solving method (*expm*), where both yielded relatively comparable outcomes. The customized implicit solvers (Radau and BDF) and *expm* also yielded comparable results when compared with the ICRP OIR Electronic Annex Distribution Set for the inhalation scenarios investigated. Consequently, the Python-based implicit ODE solvers and the Python-based algebraic solving methods yielded optimized performance for solving the metabolic problem regarding calculation speed and stability. This study establishes the foundational knowledge to further develop a robust multi-level object-oriented based framework, leveraging the dynamism in Python for improved dose reconstruction in radiation protection and nuclear medicine applications.

## Data Availability

All data that support the findings of this study are included within the article (and any supplementary information files).
